# Some Unusual Cases of Intestinal Diseases

**Published:** 1905-05

**Authors:** Henry R. Slack

**Affiliations:** President Georgia Pasteur Institute, Atlanta, and Superintendent LaGrange Sanatorium, LaGrange, Ga.


### SOME UNUSUAL CASES OF INTESTINAL DISEASES.

HENRY R. SLACK, PH.M., M.D.

President Georgia Pasteur Institute, Atlanta, and Superintendent LaGrange Sanatorium, LaGrange, Ga.*

In no department of medicine has more satisfactory progress been made in the last decade than in gastra-intestinal diseases. This is due largely to the more careful application of chemical and microscopical examinations in clinical diagnosis. No physician who values his reputation would insult the intelligence of a patient suffering from some stomach or bowel trouble by simply looking at his

* Presented to Georgia Medical Association, April 19, 1905, in Atlanta.

tongue, taking his pulse and temperature, asking a few questions and prescribing for him, as was the practice some years since. It is now necessary to give, not only a careful physical examination, but the test meal, macroscopical and microscopical examination of the feces, as well as chemical and microscopical examination of the urine. The blood count is often of value, but one would not risk his reputation on its findings alone, anemias from uncinariasis or strongyloides do not vary from secondary anemias produced by other causes.

Another reason the treatment of gastro-intestinal diseases is more successful than formerly, is that the laity as well as the profession are learning more and more to appreciate the necessity for proper diet, rest, electricity and physiological therapeutics, and not depend on medicine alone working miraculous cures.

The cases reported in this paper are not as rare as one might suppose, but until recently would not have been recognized by the general practitioner, and for this reason are here briefly presented. We will not go into the etiology or pathology of these diseases, as it is unnecessary in a paper like this; nor discuss the methods used in making the examinations, though the text-book we use and find very valuable for this work is Simon's Clinical Diagnosis.

CASE I. November 25, 1903.—L. E. P., age 33. Retired captain, U. S. V., and saw active service both in Cuba and Philippines. Complains of chronic dysentery, with fever and night sweats. Family history good. Personal history, has had usual diseases of childhood and youth. Had typhoid fever when a boy, but general health has been good until wounded in May, 1900. He then developed a cough and glands began to swell. In June, 1901, Dr. N. Senn removed the cervical glands, and after that he improved and has not been troubled with cough except when he has fresh cold. The swollen glands have never disappeared. Present troubles started in June, 1903, with dyspepsia and dysentery, which have persisted ever since, with three to six stools daily.

Examination: Poorly nourished; heart weak but no murmurs; p. 100; t. 101; slight dullness in superior lobe of right lung, and increased vocal fromitis; left lung negative; glands indurated; stomach and bowels distended with gas, very tender on pressure over abdomen, especially in region of the appendix; rectum tender, excoriated and some ulceration; weight 108 pounds; stools fluid, foul and filled with gas; microscope showed numerous tubercle bacilli in stools but none in sputum.

Treatment: Rest in bed, massage with alcohol and olive oil. Diet: raw eggs, milk with lime water, malted milk, etc. Medicines: Bismuth subnit. 2 gm. doses, liquid peptonoids with creosote, salol and borolyptol.

On this treatment he improved rapidly, stools soon had form, and were reduced to one or two daily, while he gained two pounds per week and strength in proportion.

Unfortunately he was called to Florida on business. Some time afterwards went to a sanatorium in Asheville, N. C., where he improved for a time, but relapsed and died a few months later in Knoxville, Tenn.

I report this case because it is the only one in my experience where tubercle bacilli were found in the feces and not in the sputum.

CASE II. May 19, 1904.—M. A., age 19. Schoolboy. Complains of rheumatic pains in side and shoulder with shortness of breath. Family history good. Personal history, has had usual diseases of childhood, no venereal disease, and healthy boy until four years since, when he had malaria and severe attack of rheumatism. Present trouble is sequel of grip in February, which left him with cough and swollen knee.

Examination: Poorly nourished, very anemic and sallow; heart hypertrophied with marked impulse all over cardiac area, apex beat in six interspace, mitral regurgitation; pulse 126; tempt. 101°; lungs negative except slight bronchial roughness and rapid breathing; respiration 32; R. B. C. .2700000. Plasmodium, malariæ.

He was treated with quinine, arsenis and iron, also salicylates, and improved for two weeks, as he missed his fever and pains ceased.

After eating a lot of plums without our knowledge, he had dysentery which did not yield to the usual methods, so microscopical examination of the feces was made by my assistant, Dr. V. G. Williams, and he found a very active menatode that we determined was strongyloides intestinalis. We sent specimen to Dr. H. F. Harris, of State Board of Health, and he confirmed our diagnosis. We treated this patient in the usual manner for this infection, and while he improved some, there were still a great number present when he left the sanatorium.

CASE III. April 11, 1903.—Ob. L., age 31. Male, white, factory operative. Complains of dysentery for about seven months. Family history negative. Personal history, has had usual diseases of childhood, but denies venereal diseases. Had severe attack of typhoid fever nineteen years since, but otherwise has had good health until last September, when he was attacked with dysentery and has had from six to twenty actions daily.

Examination; Very poorly nourished, emaciated man; though six feet only weighed 128 pounds; tongue sharp, round and red; heart and lungs negative; stools contain blood and mucus and amabæ coli in great numbers.

Treatment: Rest, diet, electricity and quinine gave splendid results, as in a week the stools were reduced to from four to six daily, and the second week to two or three. By the end of the eighth week he was dismissed well.

CASE IV. E. E., age 19, white, male, clerk in insurance office. Complains of dysentery since January, 1902. Family history good. Personal history, has had usual diseases of childhood and youth. General health always good until January, 1902, when he "had a severe attack of dysentery caused by eating a lot of fresh pork." This has lasted with more or less severity ever since. Several times during this period his bowels have been under control for a few days, then an exacerbation would occur and run the number of actions up to from twelve to twenty a day. He had never been free from some pain and tenderness in the bowels, and mucus and blood in small quantities had always been present in the stools.

Examination: Poorly nourished man, weight only 114 pounds; heart and lungs negative; bowels slightly distended with gas and tender on pressure, especially over the appendix; rectum raw and several small ulcers which bled when speculum was introduced; stools liquid and contained blood and mucus.

Examination showed amebæ coli in great numbers.

CASE V. J. C., age 33, farmer, white, referred by Dr. R. A. Justice. Complains of chronic dysentery for seven months. Family history negative. Personal history, has had usual diseases of childhood, but denies all venereal diseases; had pneumonia when 18; general health good up to August, 1904, when present trouble with bowels began, and from then has had from six to fourteen stools daily and had lost thirty-five pounds.

Examination: Poorly nourished man; heart and lungs negative; pulse 98; tempt. 99°; tongue red and fissured; bowels distended with gas, but no special tenderness; rectum irritated and very sensitive; stools watery, and contained some bloody mucus. Amebæ coli found.

Treatment: Of course one must vary the treatment to suit the individual case, and meet conditions as they arise, but my experience with quinine has been very satisfactory. I sometimes precede its use by cleansing the colon with hydrogen peroxide and borolyptol. Cases third and fourth have gained flesh and strength and been well for some time. Case five is still under treatment but is much improved, having gained six pounds and stools reduced to two or three daily.

The next two cases probably need an apology for being classed under the title of this paper, after the excellent work done by Drs. H. F. Harris and Claude A. Smith, of this Association, but hookworms are by no means common in this section of Georgia.

CASE VI. March 16, 1905.—H. D., referred by Dr. Chas. E. Simon, of Baltimore. Age 9. Schoolboy. Complains of being weak and nervous. Family history good. A younger brother strong and ruddy. Personal history, has never had any diseases of childhood except whooping-cough. Never sick except chills four years since, and cured of these by spending summer in Asheville. Present trouble started three years ago, and been poor and anemic ever since, but never had ground-itch until last summer. Had thirty grains of thymol in Baltimore, but did not find any worms; also male fern without effect.

Examination: Poorly nourished anemic boy; skin sallow with puffyness under eyes; tongue broad, flat and pale; heart slightly hypertrophied; lungs negative; temperature 99; pulse 88, and 100 after slight exertion; R. B. C. 3000000; no plasmodium malariæ; urine 1.020, acid, clear; feces contain ova uncinariæ, but not in great numbers.

Treated with magnesium sulphate and thymol, being careful to see that the thymol was finely powdered before putting in capsules. There were five movements and some uncinariæ passed.

CASE VII. March 30, 1905.—R. P., age 10, schoolboy from South Georgia. Complains of feeling weak and tired. Personal history, has had only whooping cough, but has never been strong; has had ground-itch lots, and a-chill since January.

Examination: Poorly nourished anemic boy; heart hypertrophied; lungs negative; pulse 98; temperature 98; liver enlarged and spleen palpable; abdomen large; legs small; R. B. C. 2710000; plasmodium malariæ; urine 1.022, acid, clear, no sugar, no albumen; feces contain ova uncinariæ in great numbers. A typical case of uncinariasis.

Treated malaria first, and when patient was stronger, thymol treatment, for hook-worms, which brought them in great numbers. Examination of feces a week later showed a few eggs still, so further treatment will be necessary.

Summary: These cases present the following unusual features: The first one tuberculosis of the intestines without active pulmonary tuberculosis. The second, strongyloides intestinalii, which had never been reported in America until Thayer, of the Johns Hopkins, reported three cases in 1901, and since only a few cases, about eight, have been reported. We found in this case that strongyloides are very resistant to all anthelmintics, as we never succeeded in securing a partheogenetic mother worm. This was also a more profound type of anemia than any with uncinariasis.

Amebic dysentery as found in three, four and five, is more common than many of us suppose. While some cases yield promptly to quinine, others require additional remedial agents, and hydrogen peroxide, borolyptol, creosote and bismuth subnitrate are very efficient.

Case six disproves the idea once prevalent that hook-worms were found only among the poorer classes and badly nourished children, as he belongs to a family of wealth and refinement. Case six agrees with the suggestion made by Dr. Claude A. Smith, that the number of uncinariæ found in a case depended on the number of attacks of ground-itch, as this boy "had ground-itch a lots" and he had lots of hook-worms.

